# Predictors and reproducibility of exercise-induced bronchoconstriction in cold air

**DOI:** 10.1186/s12890-019-0845-3

**Published:** 2019-05-16

**Authors:** Melanie Dreßler, Theresa Friedrich, Natali Lasowski, Eva Herrmann, Stefan Zielen, Johannes Schulze

**Affiliations:** 1 Division of Pulmonology, Allergy and Cystic Fibrosis, Department of Paediatric and Adolescent medicine, University Hospital Frankfurt, Theodor-Stern-Kai 7, 60590 Frankfurt am Main, Germany; 2 Institute of Biostatistics and Mathematical Modelling, Goethe-University, Frankfurt, Germany

**Keywords:** Exercise-induced bronchoconstriction, Exercise challenge in a cold chamber, Exercise challenge at an ambient temperature, Methacholine challenge test

## Abstract

**Background:**

Physical activity is an important part of life, and hence exercise-induced bronchoconstriction (EIB) can reduce the quality of life. A standardized test is needed to diagnose EIB. The American Thoracic Society (ATS) guidelines recommend an exercise challenge in combination with dry air. We investigated the feasibility of a new, ATS guidelines conform exercise challenge in a cold chamber (ECC) to detect EIB. The aim of this study was to investigate the surrogate marker reaction to methacholine, ECC and exercise challenge in ambient temperature for the prediction of a positive reaction and to re-evaluate the reproducibility of the response to an ECC.

**Methods:**

Seventy-eight subjects aged 6 to 40 years with suspected EIB were recruited for the study. The subjects performed one methacholine challenge, two ECCs, and one exercise challenge at an ambient temperature. To define the sensitivity and specificity of the predictor, a receiver-operating characteristic curve was plotted. The repeatability was evaluated using the method described by Bland and Altman (95% Limits of agreement).

**Results:**

The following cut-off values showed the best combination of sensitivity and specificity: the provocation dose causing a 20% decrease in the forced expiratory volume in 1 s (PD_20_FEV_1_) of methacholine: 1.36 mg (AUC 0.69, *p* < 0.05), the maximal decrease in FEV_1_ during the ECC: 8.5% (AUC 0.78, *p* < 0.001) and exercise challenges at ambient temperatures: FEV_1_ 5.2% (AUC 0.64, *p* = 0.13). The median decline in FEV_1_ was 14.5% (0.0–64.2) during the first ECC and 10.7% (0.0–52.5) during the second ECC. In the comparison of both ECCs, the Spearman rank correlation of the FEV_1_ decrease was r = 0.58 (p < 0.001). The 95% limits of agreement (95% LOAs) for the FEV_1_ decrease were − 17.7 to 26.4%.

**Conclusions:**

The surrogate markers PD_20_FEV_1_ of methacholine and maximal decrease in FEV_1_ during ECC can predict a positive reaction in another ECC, whereas the maximal FEV_1_ decrease in an exercise challenge at an ambient temperature was not predictive. Compared with previous studies, we can achieve a similar reproducibility with an ECC.

**Clinical trial registration:**

NCT02026492 (retrospectively registered 03/Jan/2014).

## Background

Physical activity in the context of playing and sports is an important part of life, particularly among children and adolescents, and contributes to natural development. Hence, exercise-induced bronchoconstriction (EIB) can significantly reduce the quality of life. Additionally, exercise is associated with significant health benefits, such as preventing an overweight status and obesity and reducing the risk factors of cardiovascular disease [1].

Data regarding the epidemiology of EIB range from approximately 10% in the normal population up to 90% in patients with severe asthma [2] depending on the exercise challenges used for EIB diagnosis and the challenged patient cohort [3]. For instance, a study involving 10-year-old Norwegian children showed that 8.6% of all children had EIB and that EIB occurred significantly more often in children known to have asthma (36.7%) [4]. Furthermore, the classical symptoms, such as dyspnoea, coughing or wheezing during sports, are known to have low sensitivity and specificity in predicting EIB [2, 5, 6].

Due to these reasons, a standardized test is essential for correctly diagnosing EIB. There are two different categories of bronchial provocation challenges as follows: direct and indirect challenges. In direct challenges, methacholine or histamine directly bind a smooth muscle receptor and cause bronchoconstriction. In indirect challenges, such as exercise, the inhalation of mannitol or hypertonic saline lead to increased osmolarity in the airway surfaces and consecutively to the activation of mast cells and epithelial cells, which are stimulated to release proinflammatory mediators (histamine, leukotrienes, and prostaglandins) that provoke airway smooth muscle contraction [2, 3, 7, 8].

Indirect challenges seem to be more effective in predicting EIB than direct challenges [2, 9] and are more specific for asthma, whereas direct tests are more sensitive [10]. The American Thoracic Society (ATS) recommends performing an exercise challenge in dry air, followed by serial lung function tests for the diagnosis of EIB; the cut-off value of a positive exercise challenge is an FEV_1_ decrease ≥10% [5]. Interestingly, in subjects with mild EIB, more than one exercise challenge is often required to confirm the diagnosis [11]. The reproducibility of two separate exercise challenges in combination with dry air has been reported to be 76% [12] and the intraclass correlation is good at 0.72 [13]. To diagnose EIB ATS guidelines conform we established an exercise challenge in a cold chamber (ECC) in our outpatient clinic. This method has several advantages. The patients run in a cold chamber on a treadmill without wearing a facemask for the inhalation of dry air, which is especially important in young children. In addition, the ECC simulates natural exercising at cold temperatures since participating in sports in cold environments is known to provoke EIB [14].

In addition to the exercise test, the World Anti-Doping Agency and the International Olympic Committee recommend the use of other methods to confirm the diagnosis of bronchial hyperresponsiveness (BHR) and EIB. The methacholine challenge test (MCT) is a well-established method used to assess BHR [11, 15, 16]. The sensitivity of MCT in predicting EIB ranges from 58.6 to 91.1% [11, 17]. Other recommended challenges include the inhalation of dry powder mannitol and eucapnic voluntary hyperventilation (EVH). At our department, we have broad experience with MCT [16, 18–20]; therefore, we selected this specific surrogate for comparison with the ECC.

The first aim of the present study was to determine whether MCT as a well-established test [11, 17] is a valuable predictor of EIB. The MCT as a predictor of EIB has been previously investigated in comparison with an exercise challenge at an ambient temperature and a combination of exercise and dry air [11, 17, 21–24], but not in comparison with exercise and cold air. The second aim was to determine the reproducibility of the FEV_1_ decrease and the area under the curve from 0 to 30 min (AUC_0-30min_) in detecting EIB with the ECC. Good reproducibility is a precondition for anti-asthmatic medication testing and statistical power calculations.

## Methods

### Study design

The open study consisted of four visits. Participation in the study was voluntary and written informed consent was obtained from each subject and the parents of children under the age of 18 years before starting the first visit (V1).

At V1, the inclusion and exclusion criteria were checked, the medical and medication histories were reviewed and a medical examination was performed. All children < 12 years completed the asthma control test (ACT), and all subjects ≥12 years completed the asthma control questionnaire (ACQ). All subjects performed a lung function test and an MCT.

The second visit (V2) occured between 2 weeks and 3 months after V1. The third visit (V3) occurred between 1 and 7 days after V2. During V2 and V3, an ECC was performed. During this study, we added a fourth visit (V4) to measure EIB at an ambient temperature after obtaining permission from the ethics committee.

The study was approved by the ethics committee of Goethe University. The study was registered in clinical trials under registration number NCT02026492.

### Subjects

We recruited 78 subjects aged 6 to 40 years with asthmatic symptoms while exercising with at least 1–2 training sessions per week. The children were recruited mainly from our outpatient clinic for pulmonology and allergology, and the remaining children and all adults were recruited by a public posting. Inhaled corticosteroids (ICSs) and leukotriene receptor antagonists (LTRA) were stopped from 14 days prior to participating in the study until the completion of the final study visit [25, 26]. According to the exclusion criteria, we did not include subjects with FEV_1_ < 75%, a forced vital capacity < 80%, a recent course of oral corticosteroids or other known chronic diseases or infections. Moreover, pregnancy, smoking, documented alcohol and/or drug abuse and inability to perform all study procedures were exclusion criteria for study participation.

### Pulmonary function test

Baseline pulmonary function tests were performed using a MasterScreen spirometer (CareFusion, Germany) as previously described [27].

### Methacholine challenge

The MCT was performed as previously described [16] using an Aerosol Provocation System (APS, MedicAid-dosimeter; CareFusion, Germany).

The doses of inhaled methacholine at a concentration of 16 mg/mL were increased according to the following pattern from step 1 to 5: 0.01, 0.1, 0.4, 0.8, and 1.6 mg. Two minutes after each inhalation, spirometry was performed, and the provocation was stopped under a decrease in FEV_1_ of 20% or more was reached. The individual provocation dose causing a 20% decrease in FEV_1_ (PD_20_FEV_1_) following methacholine was calculated by logarithmic interpolation using an integrated program. A PD_20_FEV_1_ < 1 mg was considered a positive reaction.

### Exercise challenge at an ambient temperature and in a cold chamber

The ECC was performed according to the ATS guidelines for the diagnosis of EIB [5] and as previously described [27]. The exercise challenge at an ambient temperature was performed similarly without using the cold chamber.

At 24 h prior to exercise challenge the subjects had to refrain from sports activities and the use of short–acting β_2_-agonists. A decrease in FEV_1_ ≥ 10% in the spirometry assessments conducted 5, 10, 15 and 30 min after running was considered as a positive reaction.

### Statistical analyses

We used GraphPad Prism 5.01 (GraphPad Software Inc., La Jolla, CA, USA), BiAS for Windows TM (version 11.0, Epsilon-Publisher, Frankfurt, Germany) and Microsoft Excel for the statistical analysis of the anonymized data.

According to the Kolmogorov-Smirnov test, the normally distributed data are expressed as the mean and standard deviation, while the non-normally distributed data are expressed as the median and minimum/maximum (min/max). If the FEV_1_ values after exercise were higher than the baseline values, they were considered a zero % decrease in FEV_1_ for better comparability with a similar study [12].

A receiver-operating characteristic (ROC) curve was plotted for the surrogate markers with significant Spearman’s rank correlations to the maximal FEV_1_ decrease in the ECC. The cut-off level with the optimal combination of sensitivity and specificity was calculated using the Youden index (sensitivity + specificity-1). The AUC reflects the accuracy of the surrogate markers in predicting a positive reaction in the ECC. Significance was set at *p* < 0.05.

The AUC was calculated as an integral from point zero to 30 min (AUC_0-30min_). The relationship between the maximum decreases in FEV_1_ after exercise and the AUC_0-30min_ was described by a Spearman’s rank correlation test.

The repeatability was evaluated using the method described by Bland and Altman [28]. Therefore, the difference between the first and second ECC is plotted against the mean of the two ECCs with the maximum FEV_1_, and the mean and SD of the differences between the two measurements along with the 95% limits of agreement (95%LOAs; mean difference ± 1.96 SDs) were calculated [28].

## Results

### Patient characteristics

Seventy-eight subjects were recruited. Two subjects did not fulfil the inclusion criteria; therefore, 76 subjects were included in the study. Five participants dropped out after V1 (one participant due to pregnancy, one participant due to a car accident, one participant withdrew consent, and two participants were lost to follow-up). Seventy-one subjects completed the first ECC on V2. Four participants dropped out between V2 and V3 as follows: one participant had a viral infection of the upper airway tract, and three participants were lost to follow-up. Sixty-seven subjects completed the second ECC at V3. Therefore, in total, 67 subjects completed both ECCs and were used for the statistical calculations.

**Fig. 1:**
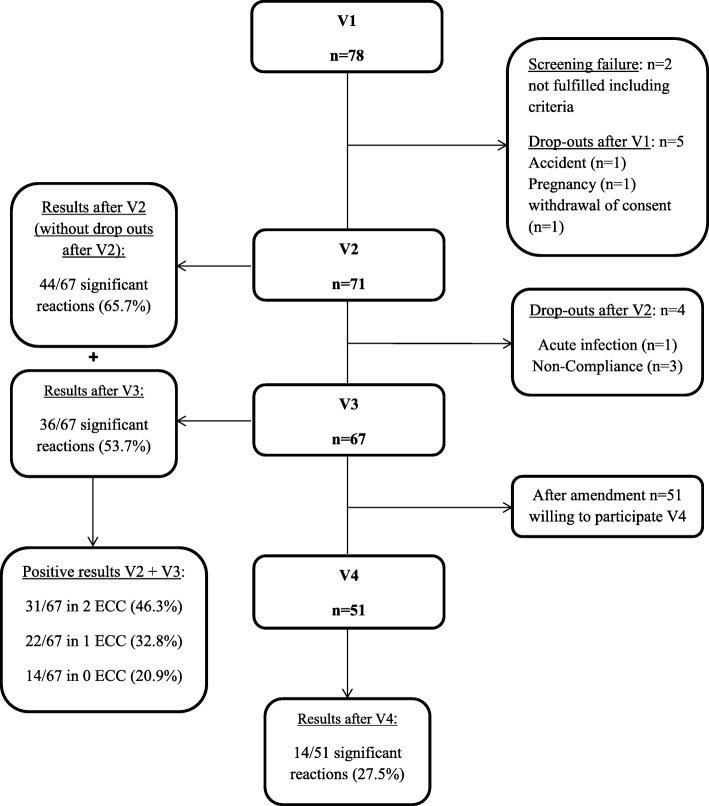
Screened subjects, included subjects, drop outs and outcome

Fifty-one of the 67 subjects (76.1%) were willing to participate in the exercise challenge at an ambient temperature (Fig. 1). Thirty-one subjects (46.3%) suffered from rhinoconjunctivitis symptoms, 14 subjects (20.9%) reported coughing during the pollen season and 21 subjects (31.3%) had physician diagnosed asthma; of these subjects, eight subjects (11.9%) took ICS on a regular basis, none of the subjects took LTRA and 35 subjects (52.2%) used short–acting β_2_-agonists as rescue medication. The mean baseline FEV_1_ value was 97.8% ± 13.0. The characteristics of the subjects and FEV_1_ values are summarized in Table 1. Regarding the FEV_1_ and FVC manoeuvres, the ATS/European Respiratory Society test criteria for acceptability and repeatability were met [29].

**Table 1:** Patient‘s characteristics

		Children	Adults	Total
		6–17 years	18–40 years	
Subjects	[*n*]	35	32	67
Female / Male	[*n*]	14/21	22/10	36/31
Age	[yr]	12.1 ± 2.9	25.6 ± 5.8	18.6 ± 8.1
Height	[m]	1.56 (1.27–1.81)	1.7 (1.55–1.9)	1.65 (1.27–1.9)
Weight	[kg]	51.4 (24.0–70.8)	65.4 (54.2–110.8)	58.1 (24.0–110.8)
FEV_1_	[%pred]	98.6 ± 12.8	97.1 ± 13.4	97.8 ± 13.0
PD_20_FEV_1_	[mg]	1.3 (0.01–2.9)	0.8 (0.01–4.5)	0.86 (0.01–4.5)
AQL		ACT (< 12 yr) 23 (19–27) *n* = 15	ACQ (≥12 yr) 0.43 (0–2.7) *n* = 50	

normally distributed data mean ± SD

not normally distributed data median and min/max

SD, standard deviation; FVC, forced vital capacity; FEV_1_, forced expiratory volume in one second; PD_20_FEV_1_ metha, provocation dose of methacholine causing a 20% drop in FEV_1_; AQL, asthma quality of life; ACT, asthma control test (cut off well controlled ≥20); ACQ, asthma control questionnaire (cut off well controlled ≤0.75), two subjects ≥12 yr did not complete the ACQ; n, number; yr., years; m, meter; kg, kilogram; %pred, %predicted; mg, milligram; ppb, parts per billion

### Overview of the reactions to the ECCs, exercise challenge at an ambient temperature and MCT

During the first ECC, 44 of the 67 subjects (65.7%) exhibited a significant reaction with a median decline in FEV_1_ among all subjects of 14.9% (0.0–46.1); during the second ECC 36 of the 67 subjects (53.7%) exhibited a reaction with a median decline in FEV_1_ among all subjects of 9.9% (0.0–52.2). The median AUC_0_-_30min_ of the entire group was a 226.6% decrease in FEV_1_/min (0.0–1045.0) after the first ECC and 149.4% decrease in the FEV_1_/min (0.0–1115.0) after the second ECC. The data of the decrease in FEV_1_ and the AUC_0_-_30min_ of the whole group and separately among the children and adults are listed in Table 2.

**Table 2:** Exercise challenge in a cold chamber

		ECC 1 (V2)			ECC 2 (V3)		
		Total *n* = 67	Children *n* = 35	Adults *n* = 32	Total *n* = 67	Children *n* = 35	Adults *n* = 32
max. FEV_1_ decrease	[%]	14.5 (0.0–64.2)	14.1 (1.5–64.2)	14.9 (0.0–46.1	10.7 (0.0–52.5)	10.8 (0.0–38.5)	9.87 (0.0–25.5)
AUC_0-30min_	[%fall FEV_1_/min]	226.6 (0.0–1045.0)	217.2 (15.6–1045.0)	264.7 (0.0–1007.0)	149.4 (0.0–1115.0)	174.5 (0.0–1115.0)	133.1 (0.0–894.4)

All values mean ± SD

*ECC* Exercise challenge in a cold chamber, *V2* Visit 2, *V3* Visit 3, *SD* Standard deviation, *FEV*_*1*_ Forced expiratory volume in one second, *AUC* Area under the curve, *p p*-value, Mann-Whitney-Test (non-parametric distribution), *p*-values: difference between child and adult group; all not significant

Thirty-one subjects (46.3%) exhibited a positive reaction with a decrease in FEV_1_ ≥ 10% from the baseline value in both ECCs; 22 subjects (32.8%) showed a positive reaction in only one of the two challenges; and 14 subjects (20.9%) did not significantly positively react in any of the challenges.

**Fig. 2:**
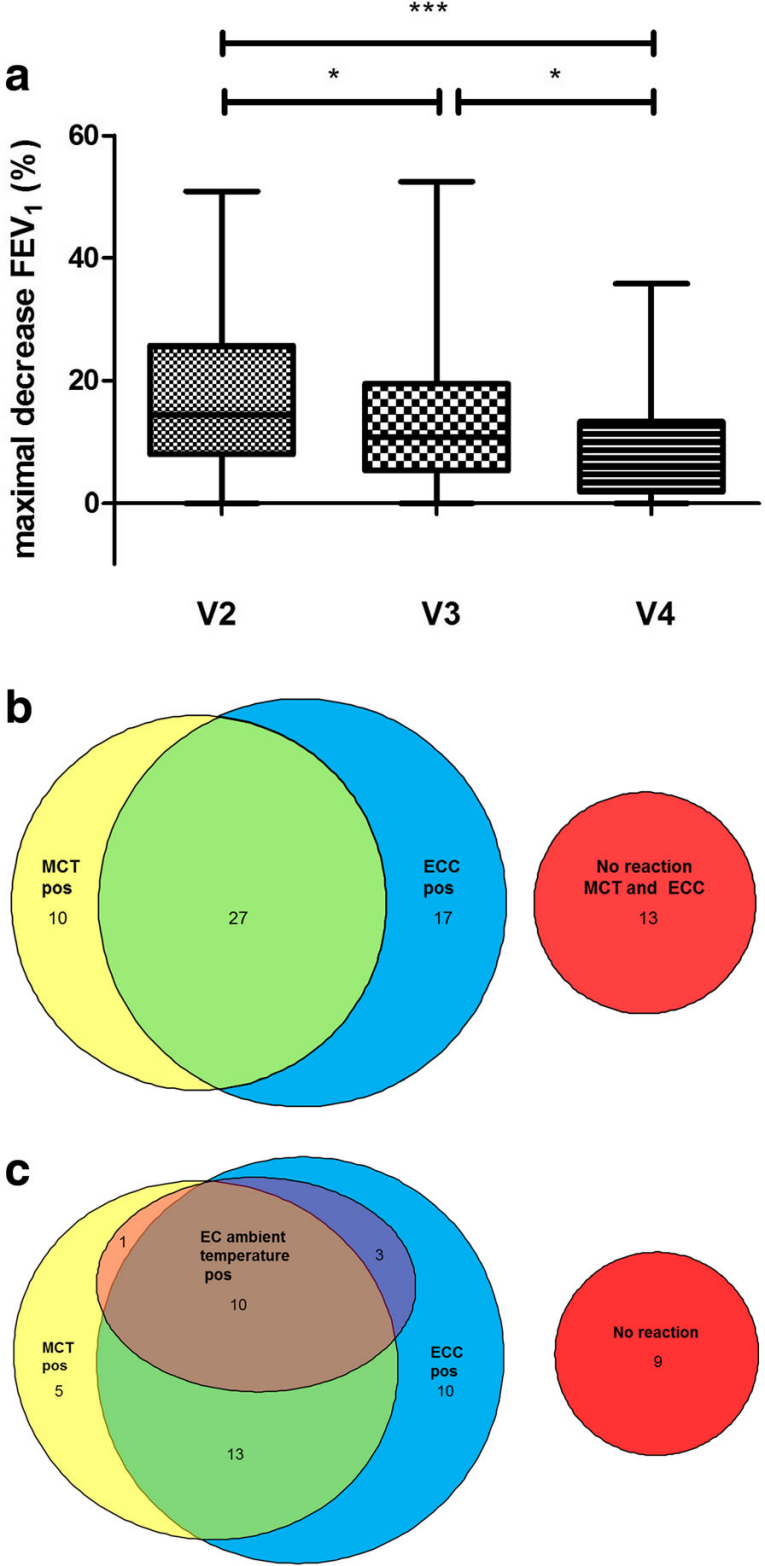
Overview of the reaction in the different bronchoprovocation tests. **a** Comparison of the maximal decrease in FEV_1_. Comparison of the maximal decrease in FEV_1_ in both ECCs (V2, V3) and the exercise challenge at the ambient temperature (V4) (*** = *p* < 0.001; * = *p* < 0.05) with median, interquartile range and min/max. **b** Overlap of the positive reactions in the first ECC and MCT (*n* = 67). **c** Overlap of the positive reactions in the first ECC, MCT and exercise challenge at an ambient temperature (*n* = 51)

During the exercise challenge at an ambient temperature, 14 of the 51 subjects (27.5%) exhibited a positive reaction with a mean decrease in FEV_1_ of 5.5% (0.0–35.8) (Fig. 1, Fig. 2 a).

The methacholine test was positive in 37 of the 67 subjects (55.2%). The overlaps in the reactions during the first ECC, MCT and exercise challenge at an ambient temperature are displayed in Fig. 2b and c.

There was a statistically significant difference between the patients with and without physician diagnosed asthma, a higher FEV_1_ decrease was observed in both ECCs (first ECC *p* < 0.01, second ECC *p* < 0.001) and a lower PD_20_FEV_1_ of methacholine was observed in the methacholine challenge (p < 0.001) but not the exercise challenge in an ambient temperature (*p* = 0.22).

### Predictors of exercise-induced bronchoconstriction in a cold chamber

The ROC curves were calculated to evaluate the sensitivity and specificity of different surrogate markers for the prediction of a positive reaction during the first ECC. As surrogate markers, the PD_20_FEV_1_ of methacholine, the maximal decrease in FEV_1_ during the second ECC and the exercise challenge at an ambient temperature were used. These three parameters were significantly correlated with the maximal decrease in FEV_1_ during the first ECC as a requirement of the ROC analysis [PD_20_FEV_1_ of methacholine: r = − 0.38 (*p* < 0.001), maximal decrease in FEV_1_ during ECC: r = 0.58 (p < 0.001), maximal decrease in FEV_1_ during exercise challenges at an ambient temperature: r = 0.39 (*p* < 0.01)].

**Fig. 3:**
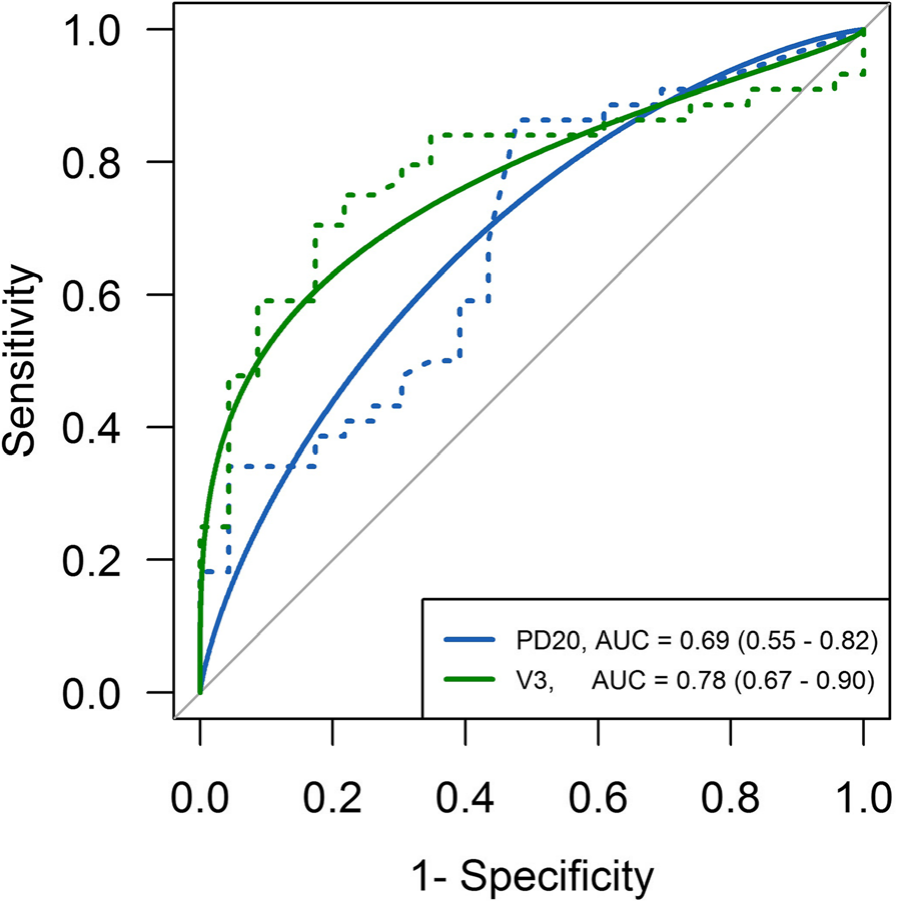
Receiver-operating characteristic curve for predicting a positive decrease in FEV_1_ after ECC. PD_20_FEV_1_ of methacholine: Optimal cut-off, 1.36 mg; sensitivity, 86%; specificity, 52%; and AUC, 0.69 (*p* < 0.05). FEV_1_ decrease during exercise challenge in a cold chamber: Optimal cut-off, 8.5%; sensitivity, 75%; specificity, 78%; and AUC, 0.78 (*p* < 0.001)

Regarding the PD_20_FEV_1_ of methacholine, the optimal cut-off value of 1.36 mg resulted in a sensitivity of 86% and a specificity of 52% (AUC 0.69, *p* < 0.05). Eleven of the 49 patients with a PD_20_FEV_1_ of methacholine below the cut-off value did not show EIB, indicating a PPV of 78%. Six of the 18 patients with a PD_20_FEV_1_ of methacholine above the cut-off value exhibited EIB, yielding an NPV of 67% (Fig. 3). In addition, after the first ECC, the optimum cut-off value for predicting a second positive ECC was 8.5% with a sensitivity of 75% and a specificity of 78% (AUC 0.78, *p* < 0.001). Five of the 39 patients with an FEV_1_ decrease over the cut-off value did not exhibit EIB in the ECC during V2, indicating a PPV of 87%. Ten of the 28 patients with an FEV_1_ decrease under the cut-off value exhibited EIB in the ECC during V2, yielding an NPV of 64% (Fig. 3).

The maximal FEV_1_ decrease in the exercise challenge at an ambient temperature could not significantly predict a positive reaction to the exercise challenge in the cold chamber [optimal cut-off decrease of FEV_1_ 5.2%, sensitivity 61%, specificity 73% (AUC 0.64, *p* = 0.13)] (data not shown).

### Reproducibility of exercise-induced bronchoconstriction in a cold chamber

**Fig. 4:**
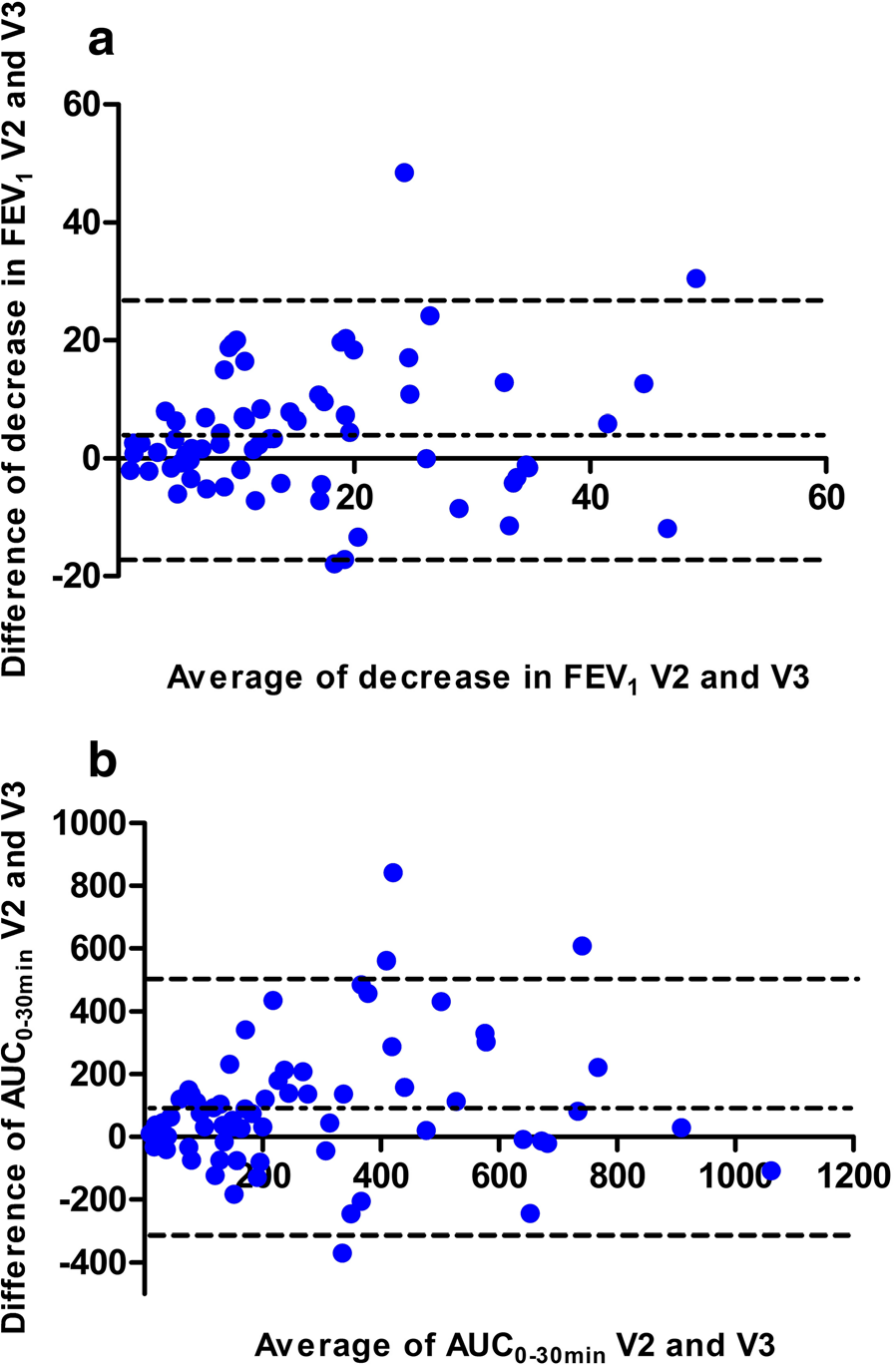
Bland and Altman plot for the maximal FEV_1_ decrease (**a**) and for AUC_0-30min_ (**b**).The difference between the first and second exercise challenges in the cold chamber is plotted against the mean of the two exercise challenges in the cold chamber for maximum FEV_1_ decrease and AUC_0-30min_, as described by Bland and Altman

In the comparison of both ECCs, the Spearman’s rank correlation of the maximal FEV_1_ decrease was r = 0.58 (*p* < 0.001) in the entire group. The agreement between the two ECCs was expressed according to the method proposed by Bland and Altman as upper and lower 95% limits of agreement (95% LOAs); regarding the maximal decrease in FEV_1_, the 95% LOAs ranged from − 17.7 – 26.4%. In Fig. 4 a, the difference between the first and second measurement is plotted against the mean of the two measurements of the maximum FEV_1_ decrease [28, 30].

A comparison of the results of the AUC_0-30min_ revealed a Spearman’s rank correlation of 0.60 (p < 0.001) and repeatability according to Bland and Altman, with 95% LOAs of − 315.7 – 504.7% (Fig. 4 b).

There was no significant difference in the mean FEV_1_ decrease after exercise between the children and adults (Table 2). Compared with the children group, we observed a better Spearman’s rank correlation between the maximal FEV_1_ decrease and the AUC_0-30min_ in the adult group. The correlation and repeatability data according to Bland and Altman (of the whole group and separately for the children and adults) are shown in Tables 3 and 4.

**Table 3:** Comparison of the two ECC - Spearman Correlation

	Total *n* = 67	Children *n* = 35	Adults *n* = 32
Maximal FEV_1_ decrease			
Spearman Correlation	0.58^**^	0.46^*^	0.77^**^
AUC_0-30min_			
Spearman Correlation	0.60**	0.34*	0.85**

FEV_1_, forced expiratory volume in one second; AUC, area under the curve

*, *p* value < 0.01;**, *p* value < 0.001

**Table 4:** Comparison of the two ECC – Bland and Altman

		Mean [%]	SD	95% CI	95% LOAs
Maximal decrease FEV_1_	Total *n* = 67	4.3	11.25	1.56–7.06	− 17.7 – 26.4
	Children *n* = 35	5.4	13.04	0.90–9.86	−20.2 – 30.9
	Adults *n* = 32	3.2	8.96	- 0.07 – 6.39	−14.5 – 20.9
AUC_0-30min_	Total *n* = 67	94.4	209.31	43.35–145.46	−315.7 – 504.7
	Children *n* = 35	116.4	253.22	28.02–204.72	− 379.9 – 612.7
	Adults *n* = 32	71.8	152.30	17.76–125.77	−226.8 – 370.3

The agreement between the two exercise challenges was expressed according to the method of Bland and Altman

Mean, mean difference of all values, SD Standard deviation of the mean difference of all values, 95% CI 95% Confidence interval of the mean difference of all values, 95% LOAs 95% Limits of agreement – range of FEV1 values in the next ECC

The 95% CI of the maximal decrease in FEV1 does not include the zero, the maximal decrease in FEV1 were averaged higher than those in the second ECC

## Discussion

The aim of the present study was to establish and to re-evaluate the combination of two indirect stimuli, i.e., exercise and cold air, using a new ECC, to achieve a higher sensitivity and reproducibility in detecting EIB. In addition, we investigated whether MCT and exercise at an ambient temperature are possible predictors of EIB using ECC as a new diagnostic tool. This analysis was performed to demonstrate for the inexperienced physicians that running at an ambient temperature has a poor sensitivity and MCT has a poor specificity in the diagnosis of EIB to underline the need for our new ECC.

In our study, the optimum cut-off value for the MCT was a PD_20_FEV_1_ of methacholine of 1.36 mg to predict a positive reaction in the ECC with a sensitivity of 85% and a specificity of 52%.

The MCT is a well-established method used to assess bronchial hyper-responsiveness (BHR) [16, 18–20]. BHR to methacholine follows a logarithmic order [31]. Therefore, the cut-off of 1.36 mg of methacholine is similar to the cut-off of 1 mg of methacholine used to predict the concentration of 8 mg/mL methacholine and is considered the usually accepted cut-off point for BHR [16]. This finding is consistent with earlier studies showing that MCT has a sensitivity of 70% and a specificity of 54.5% in predicting a positive reaction during a standardized treadmill exercise challenge in dry air in 509 patients with mild, stable asthma [11]. In a recent study involving children with asthma-like symptoms and allergic sensitization, the reactions to methacholine, mannitol, a bronchodilator test and an exercise challenge were compared [17]. The authors found BHR in 93.5% of the children with a positivity ratio of 91.1% for methacholine and 80% for mannitol. The authors concluded that a combination of both tests, i.e., a combination of a direct and an indirect challenge, increases the detection of BHR to 100%. The authors explain their findings with the fact that a direct test is more sensitive and an indirect test is more specific for EIB as both tests investigate different components of airway dysfunction [2, 9, 10, 32]. We confirm the finding that a direct challenge is more sensitive (methacholine test: sensitivity 86%, specificity 52%) and an indirect challenge is more specific (prediction decrease FEV_1_ in cold chamber: sensitivity 75%, specificity 78%). Therefore, compared with the MCT, an ECC is superior in diagnosing EIB. The ATS guidelines state that MCT is more useful for excluding a diagnosis of asthma because of its negative predictive power [26]. However, in a large multi-centre study, 73 of 163 subjects (45%) who were positive following an exercise challenge were negative following the methacholine challenge [11]. Anderson and Brannan [33] concluded that an EIB diagnosis should not be excluded on the basis of a negative MCT.

Interestingly, a maximal FEV_1_ decrease of 8.5% during the ECC significantly predicted the second positive ECC with a sensitivity of 75% and a specificity of 78%, whereas the maximal FEV_1_ decrease in an exercise challenge at an ambient temperature was not predictive. Similar results were found in patients with mild, stable asthma in a standardized exercise challenge on a treadmill while inhaling medical dry air [11]. Consistent with the literature, our study shows that an exercise challenge at an ambient temperature detects only a low percentage of subjects with EIB [15, 17]. This finding has been well known since the famous investigations performed by McFadden, who found that the severity of exercise-induced asthma varies according to the type of exercise and the environment [14]. The authors elegantly showed that running during the winter is a greater challenge for patients with asthma than running during the summer. This finding was confirmed by another study [10], which showed that combining cold air and exercise significantly increased the sensitivity of detecting exercise-induced asthma. Cold air is low in water content; thus, both cold air and dry air trigger the same mechanism, i.e. increased osmolarity in the bronchial tissue, provoking airway smooth muscle contraction [2, 3, 7, 8]. At 100% relative humidity, the water concentration in air at 37 °C is 44 mg/L; however, at − 10, 0, and + 10 °C at 100% relative humidity, the water content is 3, 5 and 9 mg/L, respectively [34]. The ATS Guidelines [5] recommend a water concentration < 10 mg/L for an exercise challenge to detect EIB. Our cold chamber has a temperature of 2 °C and 70–80% humidity, which is equivalent to a water content of 5–6 mg/L. Notably, a diagnosis of EIB has to be transferred to the real-life situation of the subjects. Of course, athletes exercising in cold and dry air are more affected by a diagnosis of EIB and need more treatment than subjects participating in sports at ambient temperature. However, importantly, the water content in our ECC corresponds to the average water content in our region during the autumn, winter and spring. Moreover, Rundell et al. [35] conclude that cold dry air and near maximal exercise intensity are critical components in an exercise challenge for the detection of EIB.

Subsequently, we investigated the reproducibility of our method on the basis of the decrease in FEV_1_ and AUC_0-30min_. The AUC summarizes the extent and duration of bronchoconstriction and, therefore, is appropriate for investigating the effect of medication on post-exercise reactions [12]. Compared with the studies conducted by Anderson et al. [12] and Dahlén et al. [13], we demonstrated similar 95% LOAs in the decrease in FEV_1_ and AUC_0-30min_ in the Bland and Altman plots of the entire group. There was no significant difference between the adults and children in the FEV_1_ decrease and AUC_0-30min_ after the ECC; however, we observed a greater variation in the children’s values. This variation leads to slightly lower correlation coefficients, wider 95% LOAs according to Bland and Altman and, consequently, lower reproducibility. This finding is consistent with a similar study in which the authors did not find significant differences in the response between adults and children, but the values for of the 95% LOAs of FEV_1_ and AUC_0-30min_ revealed a wider distribution in the values of the children [12].

Although our LOAs are similar to those reported in other studies using an exercise challenge in dry air [12, 13], they seem poorer than those in an investigation using EVH in athletic individuals (LOAs − 10.7 to 9.5%) [36]. In this study mainly healthy subjects were challenged with EVH, only 6 of 32 athletes (19%) suffered from physician-diagnosed mild asthma resulting in only 7 athletes (21.9%) exhibiting an FEV_1_ decrease ≥10% after both EVH challenges, 17 (53.1%) did not exhibit an FEV_1_ decrease ≥10% at all, and only 4 athletes exhibited an FEV_1_ decrease ≥20% after EVH challenge [36]. This prompted us to analyze our subjects with an FEV_1_ decrease < 20% (*n* = 43), < 15% (*n* = 35) and < 10% (*n* = 23) in the first ECC. The LOAs were improving ranging from − 14.3 to 17.1%, − 13.1 to 11.4% and − 11.2 to 9.3% respectively. Consequently we can conclude that the LOAs increase with the distributional width of the FEV_1_ decreases after EIB challenge and thus with the amount of subjects suffering from mild to severe BHR. Therefore, in order to compare different EIB challenge methods similar composition of the study population is essential.

The difference in the maximal decrease in the FEV_1_ values between the first and second ECC is difficult to explain. The temperature, humidity and protocol used for the treadmill exercise were identical. We speculate that the subjects were less nervous and more relaxed during the ECC, and thus, there was some type of habituation effect during the second ECC.

Another important finding is the cut-off value for a positive exercise challenge. According to the ATS guidelines, we chose an FEV_1_ decrease of ≥10% as the cut-off [5, 26, 37–40]. This cut-off was based on the mean plus two SDs of the percent decrease in FEV_1_ in healthy subjects after an exercise challenge as described in previous studies [5, 35, 41–43]. Higher cut-off values provide higher specificity and less false positive results but lower sensitivity. In a previous meta-analysis, a cut-off value of ≥13% FEV_1_ decrease with sensitivity of 62.3% and specificity of 94.2% was found to be optimal [44]. Other studies recommend using an FEV_1_ decrease ≥15% as the cut-off [5, 45], especially in field based exercise challenges [3, 46].

There are some clinical limitations to our study. First, the exercise challenge at an ambient temperature was added after the start of our study. Therefore, not all subjects underwent this challenge, and the sample size was smaller than that in the ECC analysis. Nevertheless, we could clearly show that cold air is more potent than ambient temperatures for detecting EIB. Second, it would have been preferable if the subjects practiced the ECC prior to performing it for a reproducibility test to minimize the habituation effect and not influence reproducibility. In addition, since we did not investigate control subjects, the false positive predictive value of the ECC could not be defined by our results, representing a bias and a drawback of our study.

An advantage of our method in the cold chamber is the comfortable free-run on a treadmill without wearing a facemask, which we believe is more comfortable, especially for children.

## Conclusions

In summary, the parameters PD_20_FEV_1_ of methacholine and the maximal FEV_1_ decrease in cold air were statistically significant in predicting a positive reaction during an ECC, whereas the maximal FEV_1_ decrease during an exercise challenge at an ambient temperature was not predictive.

The MCT is more sensitive and the ECC is more specific for EIB. The current and former data do not support the concept that a negative MCT excludes EIB.

We confirm that an exercise challenge at an ambient temperature detects only a low percentage of subjects with EIB [15, 17]. For confirming the diagnosis of EIB, a provocation test combining two stimuli, i.e. exercise and dry air, is essential as this approach increases the sensitivity of detecting EIB [10, 14] and is the current gold standard according to the ATS Guidelines [5].

To the best of our knowledge, the predictor PD_20_FEV_1_ of methacholine and the reproducibility of the exercise challenge were previously investigated at ambient temperatures and with the combination of exercise and dry air only but not in an ECC [11, 17, 21–24].

With our ECC, we can achieve similar reproducibility in adult patients in accordance with previous studies, using a combination of the exercise challenge and dry air. Consequently, our ECC is a good diagnostic tool and could serve as a standardized diagnostic of EIB used for the clinical testing of anti-inflammatory medications.

## Abbreviations

95% CI: 95% Confidence interval
95% LOA: 95% Limits of agreement
ACT: Asthma control test
APS: Aerosol Provocation System
AQL: Asthma quality of life
ATS: American Thoracic Society
AUC_0_-_30min_: Area under the curve at 0–30 min
BHR: Bronchial hyper-responsiveness
ECC: Exercise challenge in a cold chamber
EIB: Exercise-induced bronchoconstriction
EVH: Eucapnic voluntary hyperventilation
FEV_1_: Forced expiratory volume in 1 s
FVC: Forced vital capacity
ICS: Inhaled corticosteroid
LTRA: Leukotriene receptor antagonists
MCT: Methacholine challenge test
mg: Milligrams
NPV: Negative predictive value
PD_20_FEV_1_ of methacholine: Provocation dose of methacholine causing a 20% decrease in FEV_1_
ppb: Parts per billion
PPV: Positive predictive value
ROC: Receiver-operator characteristic
SD: Standard deviation
V1/2/3/4: Visits 1/2/3/4
